# Supplementary material for: Enhanced Electrochemical Behavior of Peanut-Shell Activated Carbon/Molybdenum Oxide/Molybdenum Carbide Ternary Composites
Source: Nanomaterials (Basel). 2021 Apr 20;11(4):1056. doi: 10.3390/nano11041056 (PMC8074364; doi:10.3390/nano11041056)
Supplement: Supplementary file 1 [file nanomaterials-11-01056-s001.zip › nanomaterials-1176000-SI.pdf]

Supplementary Materials

Enhanced Electrochemical Behavior of Peanut-Shell Activated Carbon/Molybdenum Oxide/Molybdenum Carbide Ternary Composites

Ndeye F. Sylla [1], Samba Sarr [1], Ndeye M. Ndiaye [2], Bridget K. Mutuma [1], Astou Seck [3], Balla D. Ngom [2], Mohamed Chaker [3] and Ncholu Manyala [1,*]

[1] Department of Physics, Institute of Applied Materials, SARChI Chair in Carbon Technology and Materials, University of Pretoria, Pretoria 0028, South Africa; ntoufasylla@gmail.com (N.F.S.); ssarr3112@gmail.com (S.S.); bridgetmutuma@gmail.com (B.K.M.)

[2] Laboratoire de Photonique Quantique, d'Energie et de Nano-Fabrication, Faculté des Sciences et Techniques, Université Cheikh Anta Diop de Dakar (UCAD), Dakar-Fann Dakar B.P. 5005, Sénégal; nmaty.ndiaye@gmail.com (N.M.N.); balla.ngom@ucad.edu.sn (B.D.N.)

[3] Institut National de la Recherche Scientifique Centre—Énergie Matériaux Télécommunications 1650, Boulevard Lionel Boulet, Varennes, QC J3X 1S2, Canada; astou.seck@emt.inrs.ca (A.S.); chaker@emt.inrs.ca (M.C.)

* Correspondence: ncholu.manyala@up.ac.za; Tel.: +27-12-420-3549; Fax: +27-12-420-2516

Table S1. Composition (at.%) of the PAC/MoO_2/Mo_2C ternary composites.

Peaks	Assignement	Composition (at.%)		
		PAC/MoO_2/Mo_2C-0.5	PAC/MoO_2/Mo_2C-1	PAC/MoO_2/Mo_2C-2
C 1s	C=C; C-C	62.25	49.35	52
	C-OH; O-C=O	11.51	3.22	9.54
	C-Mo_2	1.53	9.74	0.57
Mo 3d	Mo^{4+}	2.12	4.99	4.4
	Mo^{5+}	0.96	6.74	4.59
	Mo^{6+}	0.83	1.05	0.26
	Mo_2-C	1.89	2.23	0.43
O 1s	Mo-O	4.41	14.26	10.45
	O-C; O-C=O	15.86	8.42	17.77

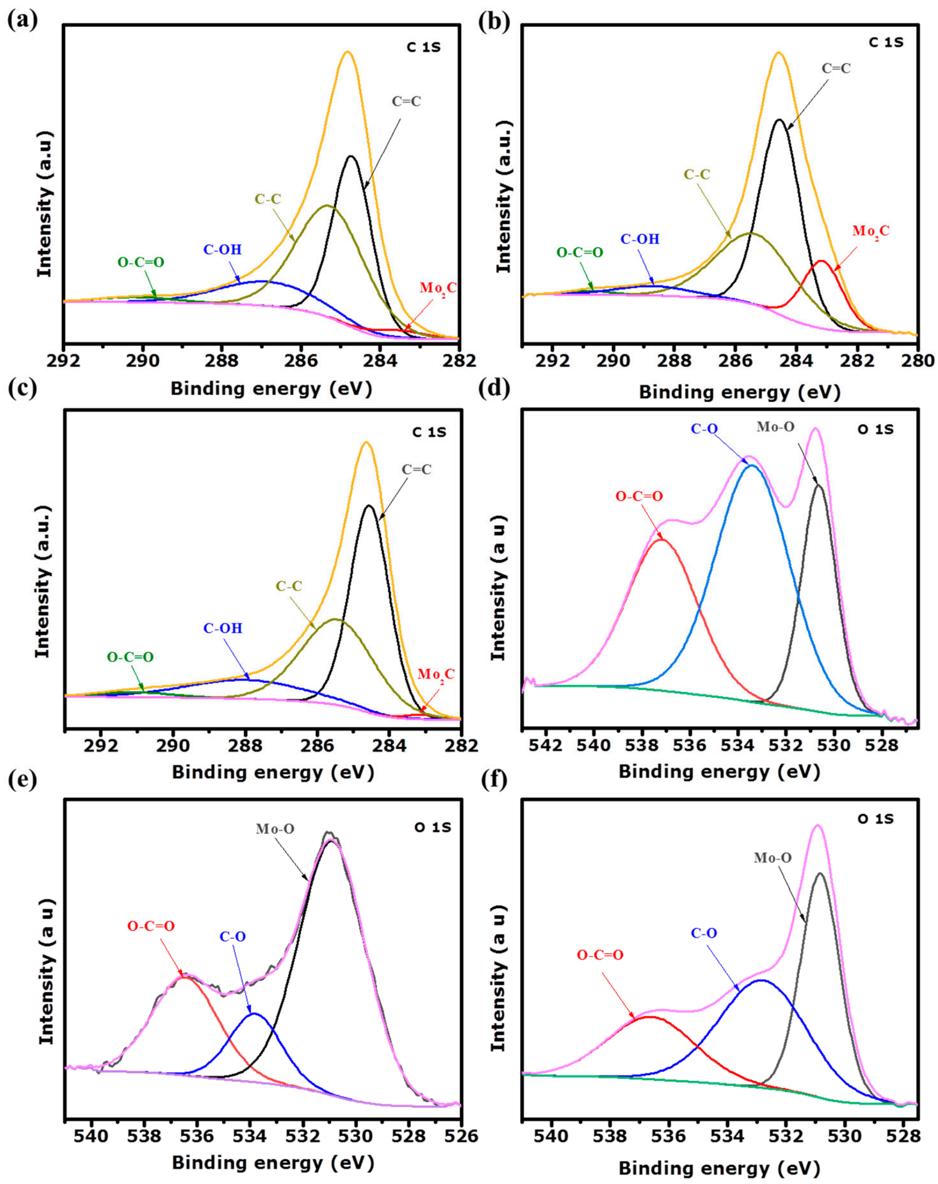

Figure S1. High resolution XPS spectra C 1s and O 1s of (**a**,**d**) PAC/MoO_2/Mo_2C-0.5 (**b**,**e**) PAC/MoO_2/Mo_2C-1 (**c**,**f**) PAC/MoO_2/Mo_2C-2 ternary composites.

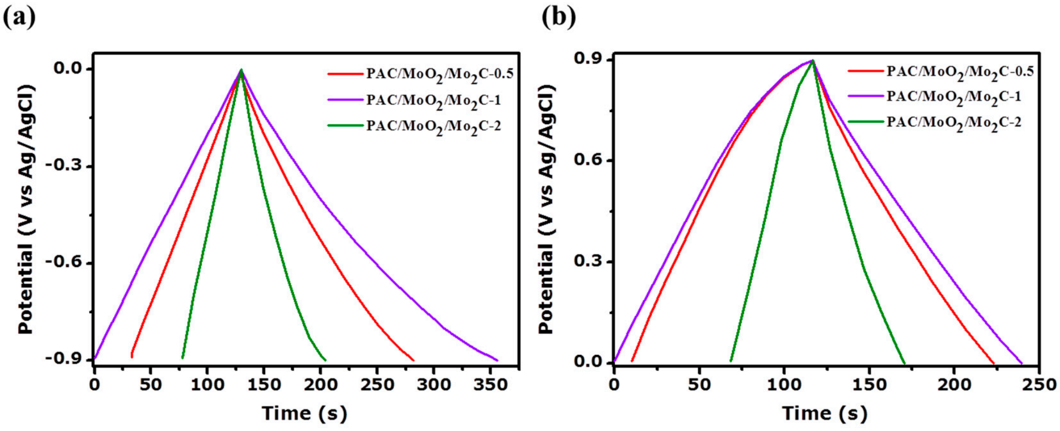

Figure S2. Galvanostatic charge-discharge curves at 1 A g^{-1} (**a**) in −0.9–0.0 V negative and (**b**) 0.0–0.9 V positive potential windows of the PAC/MoO_2/Mo_2C ternary composites in three-electrode configuration.

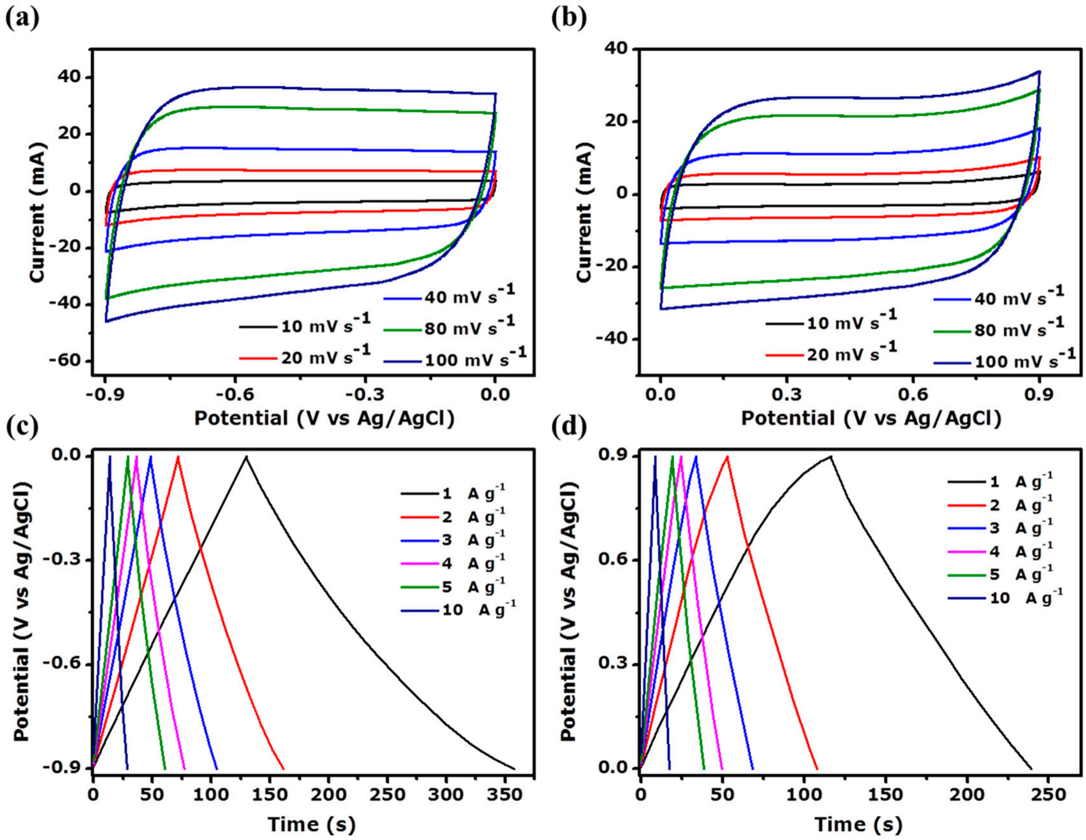

Figure S3. (**a**,**b**) CV plots at different scan rate from 10 to 100 mV s^{-1} in (−0.9–0.0 V) and (0.0–0.9 V) operating potential, (**c**,**d**) GCD curves at different specific currents ranging from 1 to 10 A g^{-1} in (−0.9–0.0 V) and (0.0–0.9 V) operating potential of the PAC/MoO_2/Mo_2C-1 ternary composite in three-electrode configuration.

Table S2. Comparison of electrochemical performance of Mo-based composite with carbon material in aqueous electrolyte.

**Materials**	**Voltage (V)**	**Electrolyte**	**Specific current (A g⁻¹)**	**Specific energy (Wh kg⁻¹)**	**Specific power (W kg⁻¹)**	**Capacitance retention (%)/cycles**	**Ref.**
PAC/MoO_2/Mo_2C-1	0–1.8	2.5 M KNO_3	1	51.8	900	83<br>25,000	This work
MoS_2/MoO_2@CNT	0–1.8	6 M KOH	0.25	11.88	2000	102.2<br>10,000	[1]
MoO_2@C/CNT	0–1.4	3 M KOH	1	25.1	704.23	87.1<br>2000	[2]
MoP/MoO_2/CNT	0–1.5	6 M KOH	0.25	31.6	190	86.5<br>10,000	[3]
MoO_2-G	−0.8–0.9	1 M Na_2SO_4	1	22.6	5774	92.5<br>1000	[4]
Mo_2C@CNT	−0.4–0.6	1 M KOH	1	50.9	500	97<br>5000	[5]
Mo-based/CNs	0–1.6	6 M KOH	1	30.8	800	97.5<br>5000	[6]

References

1. Tian, Y.; Du, H.; Zhang, M.; Zheng, Y.; Guo, Q.; Zhang, H.; Luo, J.; Zhang, X. Microwave synthesis of MoS_2/MoO_2@CNT nanocomposites with excellent cycling stability for supercapacitor electrodes. *J. Mater. Chem. C* **2019**, *7*, 9545–9555, doi:10.1039/c9tc02391g.
2. Si, H.; Sun, L.; Zhang, Y.; Wu, L.; Zhang, Y.; Zhang, Y. Enhanced pseudocapacitive energy storage properties of budding-branch like MoO_2@C/CNT nanorods. *Dalt. Trans.* **2020**, *49*, 1637–1645, doi:10.1039/c9dt04391h.
3. Tian, Y.; Sarwar, S.; Zheng, Y.; Wang, S.; Guo, Q.; Luo, J.; Zhang, X. Ultrafast microwave manufacturing of MoP/MoO_2/carbon nanotube arrays for high-performance supercapacitors. *J. Solid State Electrochem.* **2020**, *24*, 809–819, doi:10.1007/s10008-020-04524-2.
4. Thangappan, R.; Arivanandhan, M.; Kalaiselvam, S.; Jayavel, R.; Hayakawa, Y. Molybdenum Oxide/Graphene Nanocomposite Electrodes with Enhanced Capacitive Performance for Supercapacitor Applications. *J. Inorg. Organomet. Polym. Mater.* **2018**, *28*, 50–62, doi:10.1007/s10904-017-0699-1.
5. Hussain, S.; Rabani, I.; Vikraman, D.; Feroze, A.; Karuppasamy, K.; Haq, Z. ul; Seo, Y.-S.; Chun, S.-H.; Kim, H.-S.; Jung, J. Hybrid Design Using Carbon Nanotubes Decorated with Mo_2C and W_2C Nanoparticles for Supercapacitors and Hydrogen Evolution Reactions . *ACS Sustain. Chem. Eng.* **2020**, *8*, 12248–12259, doi:10.1021/acssuschemeng.0c04022.
6. Yang, W.; Deng, B.; Hou, L.; Wang, T.; Tian, J.; Wang, S.; Li, R.; Yang, F.; Li, Y. Sulfur-fixation strategy toward controllable synthesis of molybdenum-based/carbon nanosheets derived from petroleum asphalt. *Chem. Eng. J.* **2020**, *380*, 122552, doi:10.1016/j.cej.2019.122552.
